# Grapevine VpPR10.1 functions in resistance to *Plasmopara viticola* through triggering a cell death‐like defence response by interacting with VpVDAC3

**DOI:** 10.1111/pbi.12891

**Published:** 2018-03-08

**Authors:** Hui Ma, Gaoqing Xiang, Zhiqian Li, Yuting Wang, Mengru Dou, Li Su, Xiao Yin, Ruiqi Liu, Yuejin Wang, Yan Xu

**Affiliations:** ^1^ State Key Laboratory of Crop Stress Biology for Arid Areas College of Horticulture Northwest A&F University Yangling Shaanxi China; ^2^ Key Laboratory of Horticultural Plant Biology and Germplasm Innovation in Northwest China Ministry of Agriculture Northwest A&F University Yangling Shaanxi China

**Keywords:** grapevine, *Plasmopara viticola*, PR10, ROS, voltage‐dependent anion channel, *Vitis pseudoreticulata*

## Abstract

As one of the most serious diseases in grape, downy mildew caused by *Plasmopara viticola* is a worldwide grape disease. Much effort has been focused on improving susceptible grapevine resistance, and wild resistant grapevine species are important for germplasm improvement of commercial cultivars. Using yeast two‐hybrid screen followed by a series of immunoprecipitation experiments, we identified voltage‐dependent anion channel 3 (*VDAC3*) protein from *Vitis piasezkii* ‘Liuba‐8’ as an interacting partner of *VpPR10.1* cloned from *Vitis pseudoreticulata* ‘Baihe‐35‐1’, which is an important germplasm for its resistance to a range of pathogens. Co‐expression of *VpPR10.1*/*VpVDAC3* induced cell death in *Nicotiana benthamiana,* which accompanied by ROS accumulation. *VpPR10.1* transgenic grapevine line showed resistance to *P. viticola*. We conclude that the VpPR10.1/VpVDAC3 complex is responsible for cell death‐mediated defence response to *P. viticola* in grapevine.

## Introduction

Grape is one of the world's oldest cultivated fruit crops, with the increase in population and rising standards of living, consumption of grapes and grape products (notably wine) continues to grow. Increasing production areas and efficiency makes commercial grapes the second highest value fruit crop in the world, according to FAO report (2016). Downy mildew is one of the most serious biotrophic parasite diseases in grapes, it is caused by *Plasmopora viticola* and mainly infects the green tissues (Ingram, 1981). *P. viticola* is the foremost threat to the wine grape industry in most parts of the world (Vercesi *et al*., 1999). Chinese wild grape varieties are important gene resources for improving disease resistance of susceptible cultivars, and a number of studies have shown that genes derived from Chinese wild grapes play important roles in grape disease resistance (Han *et al*., 2016; Xu *et al*., 2014; Yu *et al*., 2013a). Many important grape cultivars are severely compromised by downy mildew, so improvements in disease resistance are urgent need for grapevine breeders. Wild grapes present abundant genetic sources of disease resistance for grape breeding. For example, resistance against downy mildew found in wild American species has been successfully integrated into commercial grape cultivars (Eibach *et al*., 2009; Gessler *et al*., 2011; Jiao *et al*., 2016).

Pathogenesis‐related (PR) proteins served as various roles of defence in plants (Loon and Kammen, 1968; van Loon *et al*., 2006). Increasing evidences show that PR10 (pathogenesis‐related protein 10) has distinct functions in plant developmental, the secondary metabolism and in bacteriostatic action (Choi *et al*., 2012; Hashimoto *et al*., 2004; Liu and Ekramoddoullah, 2006; McGee *et al*., 2001; Zhou *et al*., 2002). PR10s are highly conserved among a range of plants, and there are seventeen PR10s in *Vitis vinifera* (Lebel *et al*., 2010), although the exact biological functions of PR10 have not yet been clarified, many studies show PR10 proteins are induced by biological stresses, at the same time PR10 proteins enhance the resistance to viruses (Loon, 1997), bacteria (Breda *et al*., 1996) and fungi (Swoboda *et al*., 1995). In grape, VpPR10.2 shows nuclease activity and contributes to host resistance to *P. viticola* (He *et al*., 2013). Besides grapevine, *avirulent Xanthomonas campestris* pv. *vesicatoria* (*Xcv*) induced *PR10* associated with the hypersensitive response in pepper and *Nicotiana benthamiana*, overexpression of pepper *PR10* and *LRR1* in *Arabidopsis thaliana* showed resistance to *Hyaloperonospora arabidopsidis* and the cytosolic *LRR*/*PR10* complex in pepper is responsible for cell death‐mediated defence reaction (Choi *et al*., 2012).

There are two layers of immune defence systems in plants: the first layer involves pattern‐recognition receptors which detect the pathogen/microbe‐associated molecular patterns called PAMP/pattern‐triggered immunity (PTI). These offer a broad spectrum of resistance in terms of a nonspecific immune response (Dodds and Rathjen, 2010; Jones and Dangl, 2006). To overcome the pathogen invasion, plants use another layer of defence called effector‐triggered immunity (ETI) (Koistinen *et al*., 2002; Wang *et al*., 2009). Resistance (R) proteins recognize pathogens and induce hypersensitive response (HR), and HR reaction induces programmed cell death, which limits the ability of the pathogenic bacteria to reproduce (Bittel and Robatzek, 2007).

Voltage‐dependent anion channel (VDAC) proteins are among the most abundant proteins found in the mitochondrial outer membrane (Benz, 1994; Colombini, 1979), and these are important for regulating material and energy exchange between mitochondria and the cytoplasm. Apoptosis factors also exist in mitochondria, such as cytochrome c, the apoptosis factors released into the cytoplasm to induce cell death (Kroemer *et al*., 2007). In plants, VDACs also mediate PCD resulting from biotic and abiotic stresses (Benz, 1994; Colombini, 1979; Sangmin *et al*., 2009). The expression of *AtVDAC1, AtVDAC2, AtVDAC3* and *AtVDAC4* is up‐regulated in response to *pst* DC3000. This suggests that VDACs play an active role response to biotic stress (Robert *et al*., 2012). It has been shown that AtVDAC3 specifically binds to the kinesin motor protein KP1 to control seed germination at low temperatures (Yang *et al*., 2011). *AtVDAC3* can also interact with *Atthioredoxinm2*, the overexpression of *AtVDAC3* increased H_2_O_2_ accumulation, while overexpression of *AtTrxm2* weakened H_2_O_2_ generation, and hence, they play opposing roles in stress responses involving ROS signalling (Zhang *et al*., 2015). Recent studies have shown the influential role of *metacaspases* in plant PCD, including pathogen infection (Hao *et al*., 2007; Hoeberichts *et al*., 2003), stimulation of H_2_O_2_ (He *et al*., 2008) and plant development (Suarez *et al*., 2004). Plant *metacaspases* are thought to be distantly related to animal caspases and can be classified as type I and type II (Lam, 2004; Uren *et al*., 2000). Studies have also shown that two *metacaspases AtMCl* and *AtMC2* of *Arabidopsis* play positive and negative regulating roles, respectively, in plant cell death (PCD) (Coll *et al*., 2010). Although some progress has been made in characterizing the *metacaspases* (Lam and Zhang, 2012; Tsiatsiani *et al*., 2011; Vercammen *et al*., 2007; Zhang and Lam, 2011), a detailed overview of their biochemical properties is still lacking.

In our previous study, *VpPR10.1* was cloned from Chinese wild grape *V. pseudoreticulata* and VpPR10.1 contributes to defence reactions in grape (Xu *et al*., 2014). In this study, we show that *VpPR10.1* transgenic grapevine have elevated ROS production after *P. viticola* inoculation. We demonstrate the ability of VpPR10.1 promoting immunity to *P. viticola* through a pathway involving with VpVDAC3. They work synergistically in a cell death‐like defence response to enable resistance to *P. viticola* in grape.

## Results

### VpPR10.1 interacts with VpVDAC3

**Figure 1:**
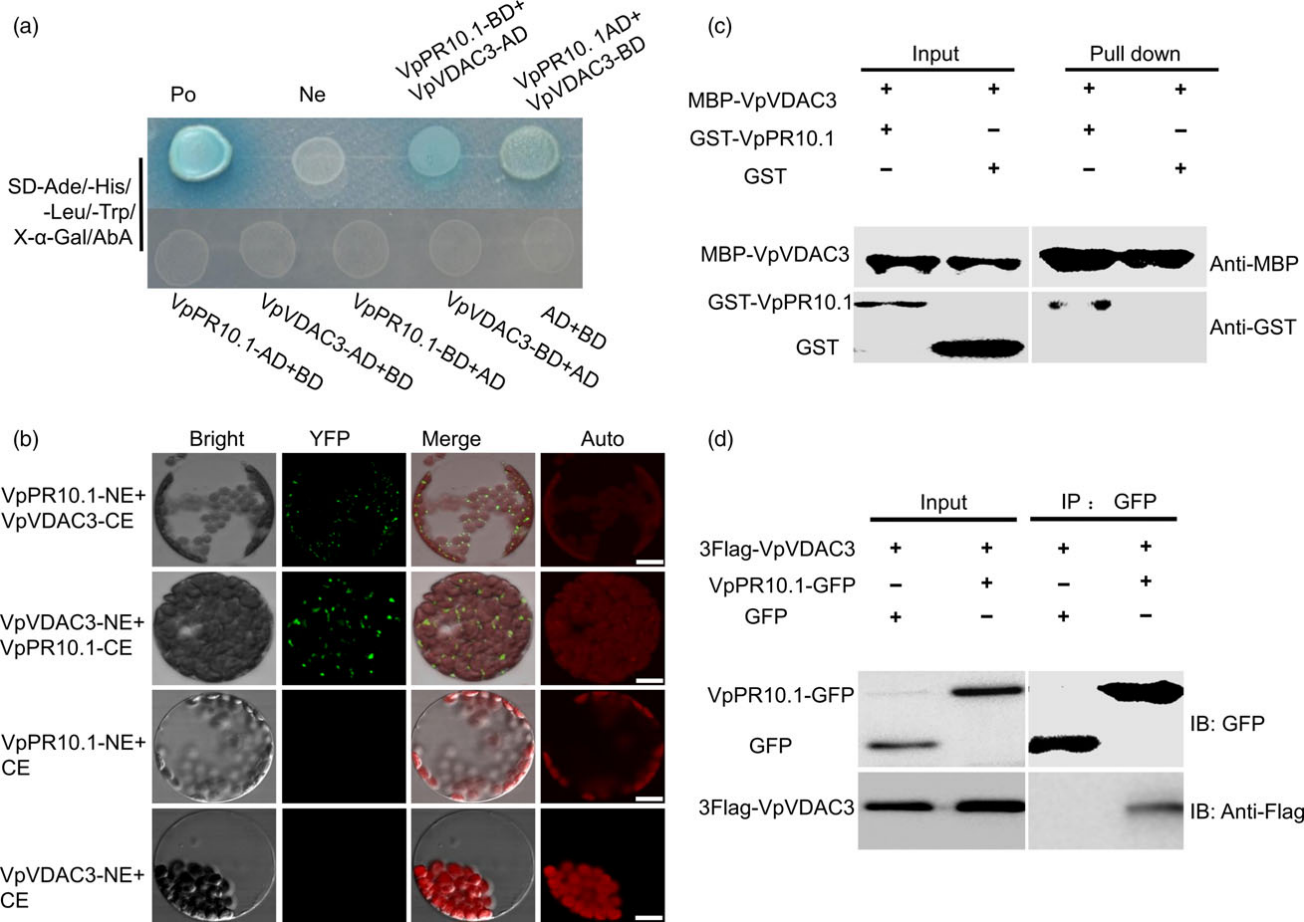
VpPR10.1 interacts with VpVDAC3. (a)Yeast two‐hybrid assay. pGBKT7 or pGADT7 plasmid containing *VpPR10.1* and *VpVDAC3* was transformed into Y2H Gold. Combinations of the (AD/T) with BD/p53 and BD/Lam were used as positive and negative controls. (b) Bimolecular fluorescence complementation (BiFC) assay *in vivo*. Merged fluorescent and visible light images were taken. Bars = 10 μm. Auto, chloroplast auto‐fluorescence. (c) Pull‐down assay. The presence or absence of each protein in the final mixture is indicated as + or −, respectively. (d) Co‐IP and immunoblotting (IB) of GFP/Flag‐*VpVDAC3* and *VpPR10.1*‐GFP/Flag‐*VpVDAC3* were co‐expressed in *Nicotiana benthamiana* leaves. Flag antibodies were used for the detection of immunoprecipitated VpPR10.1‐GFP and Flag‐VpVDAC3.

We previously obtained the *VpPR10.1* from *V. pseudoreticulata* ‘Baihe‐35‐1’, a wild grape that shows resistance to multiple grapevine diseases (Wan *et al*., 2007; Wang *et al*., 1995; Xu *et al*., 2014). We used yeast two‐hybrid system to screen interacting partners to identify the resistance mechanism of VpPR10.1. Target proteins were baited from a library construct from resistant Chinese wild grape *V. piasezkii* ‘Liuba‐8’ after *P. viticola* infection. Among fifty‐six positive clones we obtained (Table S1), four of these positive clones contained the same cDNA that encodes VpVDAC3 (voltage‐dependent anion channel), which was selected as the target protein (Figure 1a). To test the interaction in living plants, bimolecular fluorescence complementation (BiFC) assay (Bracha *et al*., 2004; Walter *et al*., 2004) was carried out. N‐terminal (NE) and C‐terminal (CE) fragments of yellow fluorescent protein (YFP) were connected to VpPR10.1 and VpVDAC3, and coupled constructions were expressed in tobacco protoplasts. Fluorescence was detected in the combination of VpPR10.1‐NE/VpVDAC3‐CE and VpVDAC3‐NE/VpPR10.1‐CE, but not in the control samples (Figure 1b). As shown in Figure 1c, recombinant protein GST‐*Vp*PR10.1 but not unmodified protein GST was bond by MBP‐*VpVDAC3*. Next, we tested the interaction between VpPR10.1 and VpVDAC3 using CO‐IP. We found that 3Flag‐VpVDAC3 co‐precipitated with VpPR10.1‐GFP but not with the control GFP (Figure 1d). Taken overall, we conclude that VpPR10.1 interacts directly with VpVDAC3.

### The localization of *VpPR10.1* and *VpVDAC3 in vivo*

**Figure 2:**
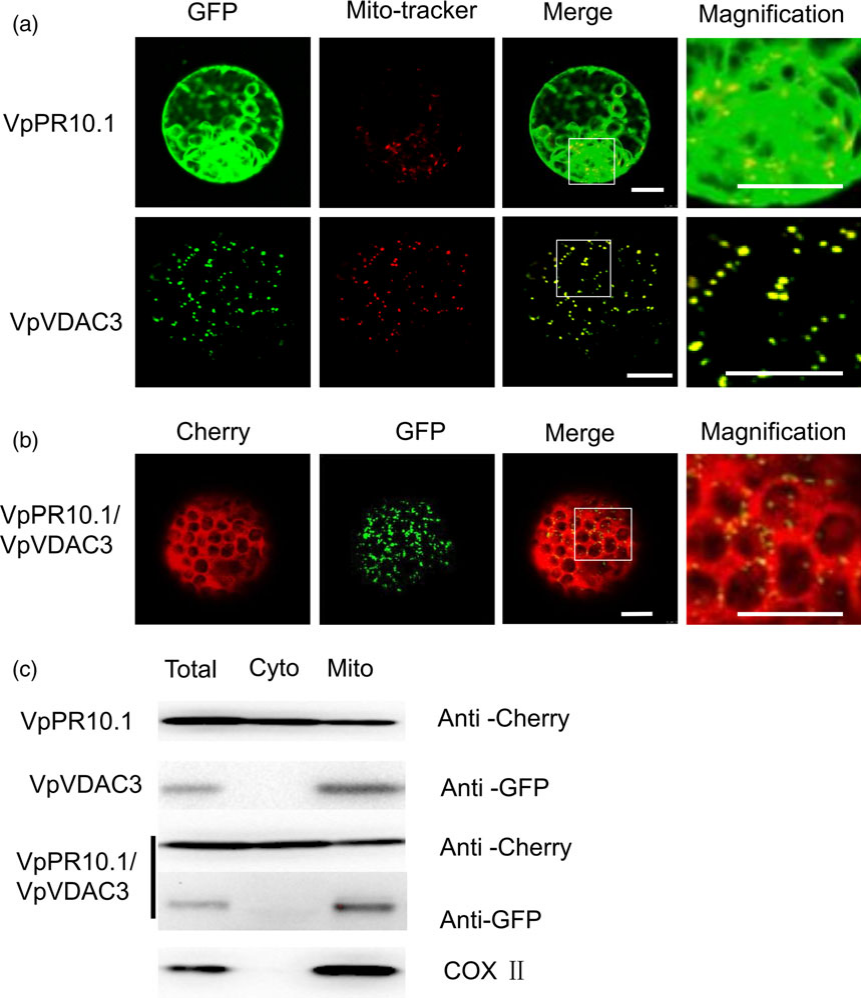
Subcellular localization of *VpPR10.1* and *VpVDAC3*. The visible fluorescence indicates gene localization in *Nicotiana benthamiana* protoplasts. (a) Localization of *VpPR10.1*‐GFP,* VpVDAC3*‐GFP. Mito‐Tracker Red (Invitrogen) was stained to mark mitochondria. Bars = 10 μm. (b)Co‐localization of the *VpPR10.1* and *VpVDAC3* in *N. benthamiana* protoplasts transformed with the couple constructs of cherry‐*VpPR10.1* and *VpVDAC3*‐GFP. Bars = 10 μm. (c) Mitochondria were extracted from transformed protoplasts and detected by immunoblotting. Total, no separated protein; Cyto, nonmitochondrial protein; Mito, mitochondrial enriched protein. COX II was present as mitochondrial marker protein.

Our previous research found that VpPR10.2 is located in the host cell dynamically (He *et al*., 2013). In our study, we found that VpPR10.1‐GFP diffusely localized in the cytoplasm of *N. benthamiana* protoplasts (Figure 2a). At the same time, *VpVDAC3* targeting to mitochondria was confirmed by co‐staining with Mito‐tracker Red which served as a mitochondrial‐specific reagent. To gain insight into the complex interaction, cherry‐VpPR10.1 and VpVDAC3‐GFP were co‐transformed (Figure 2b), VpVDAC3‐GFP can colocalized with VpVDAC3‐GFP. As an independent component of potential co‐localization of VpPR10.1 and VpVDAC3, we extracted mitochondria and analysed by Western blotting. As seen in Figure 2c, VpVDAC3 and VpPR10.1 were both detected in mitochondrial fractions.

### Transient co‐expression of *VpPR10.1* accelerated ROS production induced by *VpVDAC3*

**Figure 3:**
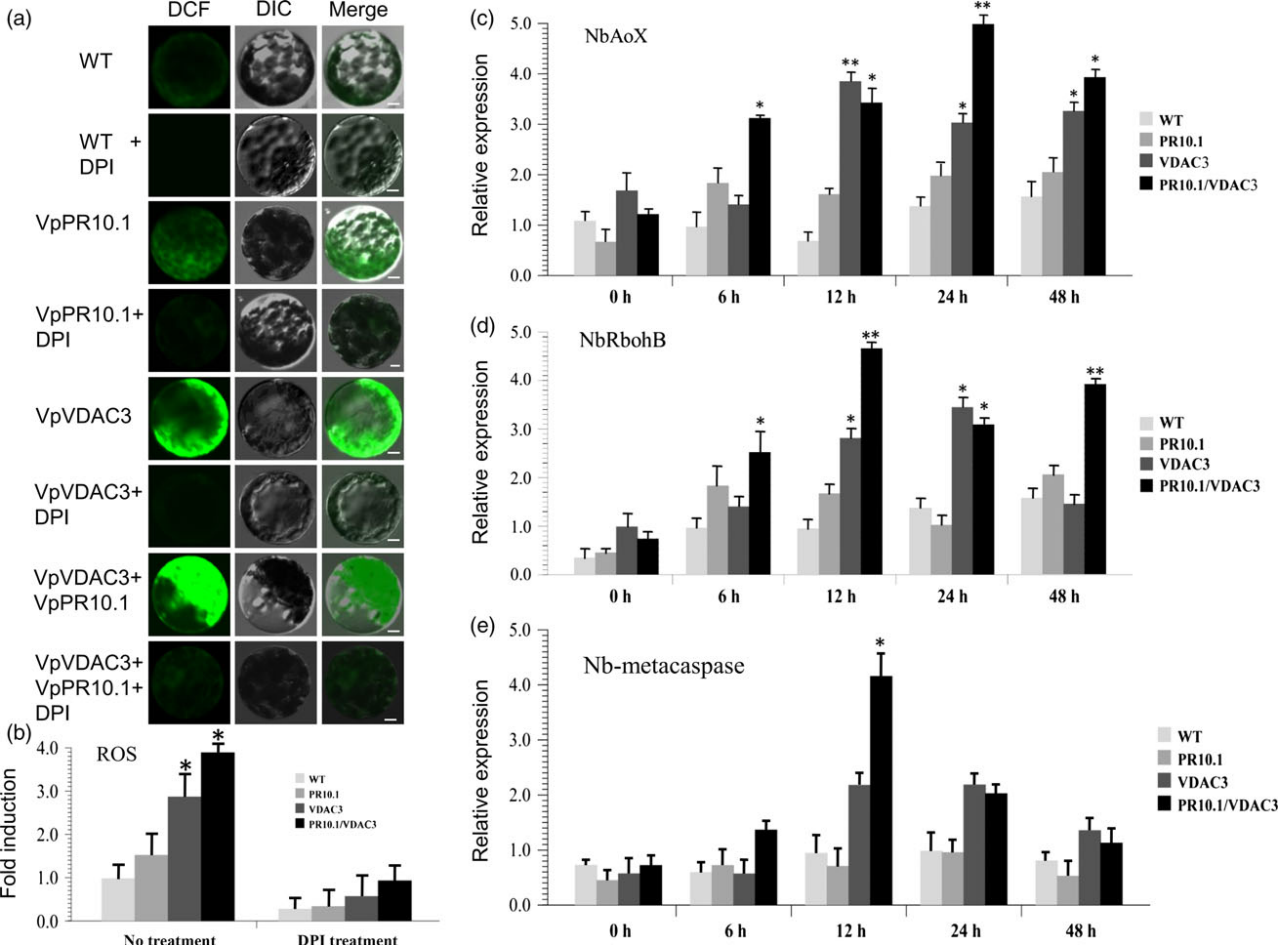
ROS Detection of Transient Expression of *VpVDAC3*,* VpPR10.1*,* VpVDAC3*/*VpPR10.1* in *N. benthamiana*. Before the separation of protoplasts, Flag‐VDAC3, Flag‐VpPR10.1 and Flag‐VpVDAC3/Flag‐VpPR10.1 were transiently expressed by *Agrobacterium* infiltration in *N. benthamiana* leaves. (a) Protoplasts isolated from the indicated transient expression leaves, and protoplast lines were marked by DCFH‐DA. The green fluorescence represents intracellular ROS level; (b) Each sample was accompanied by pretreatment of ROS inhibitor DPI. Bars = 10 μm. (c, d, e) Quantification of RT‐PCR analysis of ROS‐related genes *NbAOX*,* NbRbohB*,* NbAPX*; values represent means and SEs of three biological replicates. * and ** indicate *P* < 0.05 and *P* < 0.01, compared to GFP control (*t*‐test).

There is a consensus view that excessive ROS in cells can lead to damage and is closely associated with cell death (Carlisle *et al*., 2000; Kim *et al*., 2006; Son *et al*., 2010). *VDAC* has been identified as an important mediator of apoptosis closely related to oxidative signals (Madesh and Hajnóczky, 2001). Overexpression of *VDAC1* strengthens the ROS production and triggers apoptosis (Simamura *et al*., 2006). DCFH‐DA becomes irreversibly fluorescent by a distinctive reaction with ROS (Choi *et al*., 2010; Kim *et al*., 2003, 2006). To assess the effect of VpVDAC3 on ROS, we separated the protoplast from the transient expressed leaves of indicated genes and visualized ROS using DCFH‐DA. The *Agrobacterium tumefaciens* carried recombinant plasmids (Flag‐*VpVDAC3*, Flag‐*VpPR10.1* and Flag‐*VpVDAC3*/Flag‐*VpPR10.1*) were transiently expressed in *N. benthamiana* before the separation of protoplasts. In the protoplast fluorescent‐dye assay, fluorescence indicates the production of ROS (Figure 3a). An increase in fluorescence was observed in protoplasts containing *VpVDAC3,* and this effect was enhanced by the co‐expression of *VpPR10.1*. This indicates *VpPR10.1* enhanced the ability of *VpVDAC3*’ to induce ROS production. We also treated protoplasts with DPI as a NADPH oxidase inhibitor, fluorescence in the DPI‐pretreated protoplasts was lower than that in the untreated ones, and DPI decreased the ROS accumulation in the protoplasts (Figure 3b). These results suggest that NADPH oxidase is involved in the ROS burst that caused by VpVDAC3.

Also, ROS‐related genes were confirmed by RT‐PCR after infiltration, as *AOX* plays an important role during the response to stress, we examined the *NbAOX* transcript levels. As shown in (Figure 3c), co‐expression of *VpPR10.1* and *VpVDAC3* resulted in induction of *NbAOX* by 3.5‐fold compared with the negative control (GFP). *NbRboh* expression was also strongly induced by *VpPR10.1* and *VpVDAC3* co‐expression (Figure 3d), and the ascorbate peroxidase *NbAPX* was up‐regulated too (Figure 3e).These results indicate VpPR10.1 positively regulate the ROS activity through VpVDAC3 in plant cells.

### Depolarization of mitochondria membrane by transient expression of *VpVDAC3*/*VpPR10.1* in *N. benthamiana*

**Figure 4:**
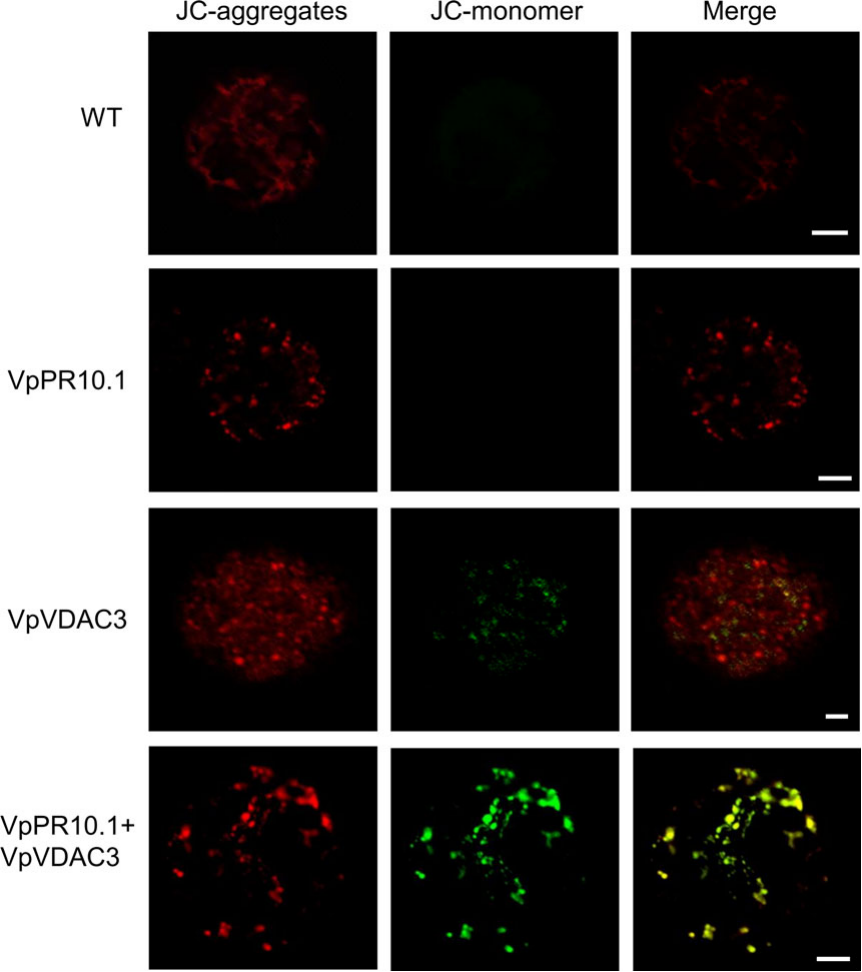
Depolarization of Mitochondria by Transient Expression *VpVDAC3*/*VpPR10.1* in *Nicotiana benthamiana*. Mitochondrial depolarization. Living protoplasts were separated from *N. benthamiana* leaves that transiently expressed indicated genes. Decreased mitochondria membrane polarization results in the formation of JC‐1 aggregate to monomer which results in shifts of red/orange to green. Bars = 10 μm.

Loss of mitochondrial membrane potential is an early step in apoptosis, and we used a marker of mitochondrial membrane potential dye JC‐1 to evaluate the effect of VDAC or PR10 overexpression on mitochondrial membrane potential in *N. benthamiana*. Protoplasts were isolated from the transient expressed *N. benthamiana* leaves, protoplast lines were marked by JC‐1, and JC‐1 changes colour from red/orange to green as the membrane potential decreases (Cossarizza *et al*., 1993; Salvioli *et al*., 1997). In Figure 4, the green fluorescence represents mitochondrial membrane potential loss, and *VpVDAC3*/*VpPR10.1* together induce higher decreases of mitochondrial membrane potential.

### Transient co‐expression of VpPR10.1 and VpVDAC3 promotes ROS production and cell death in *N. benthamiana*

**Figure 5:**
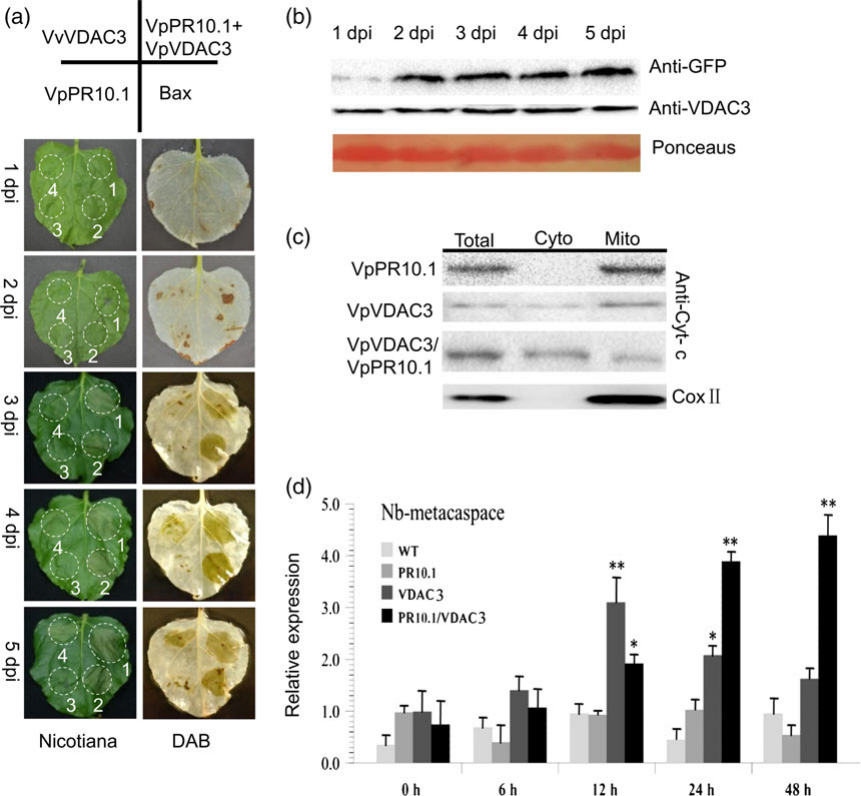
Transient Expression of *VpVDAC3* or *VpVDAC3*/*VpPR10.1* in *Nicotiana benthamiana* induces cell death. Phenotypic and physiological analyses of *VpPR10.1* and *VpVDAC3* following DAB staining. (a) Phenotypes of *VpVDAC3*,* VpPR10.1*,* VpVDAC3*/*VpPR10.1* and *Bax* were all injected into one *N. Benthamiana* leaves. The bluish colour of DAB staining represents accumulated H_2_O_2_. (b) Visualization of VpVDAC3, VpPR10.1 protein by Western blotting in *Agro*‐infiltration *N. benthamiana* leaves. Ponceaus staining stand for control loading. (c) Western blot for detecting cytochrome c after expression with indicator gene in *N. Benthamiana* leaves. Total, nonseparated protein; Cyto, nonmitochondrial protein; Mito, mitochondrial enriched protein. COX II was present as mitochondrial marker protein. (d) Quantification of RT‐PCR analysis of cell death inducing gene *Nb‐MCA1* expression. Values mean ± SE (*n* = 3) of three independent biological repeats. Asterisks indicate significant difference between each gene and GFP control, *P < 0.05; **P < 0.01 (*t*‐test).

Overexpression of VDAC triggers cell death in a diverse range of organisms including yeast, rice, fish, tobacco, mouse and humans (Godbole *et al*., 2003, 2013; Shoshan‐Barmatz *et al*., 2010; Zaid *et al*., 2005). To determine the biological function of *VpVDAC3* and *VpPR10.1*, we evaluated the effect of co‐expression on cell death (Figure 5a). Expression of VpPR10.1 and VpVDAC3 was confirmed by Western blot (Figure 5b) and 3,3‐diaminobenzidine (DAB) staining (Figure 5a). Transient expression of *VpPR10.1* did not trigger an obvious reaction in *N. benthamiana* (Figure 5a). However, co‐expression of *VpPR10.1*/*VpVDAC3* triggered enhanced cell death than *VpVDAC3* (Figure 5a). The sites of leaves infiltrated by *VpPR10.1*/*VpVDAC3* showed enhanced H_2_O_2_ than *VpVDAC3* alone too (Figure 5a), and *VpPR10.1* positively regulated the cell death responses induced by *VpVDAC3*. To confirm that cell death in *N. benthamiana* leaves after infiltration, we assayed the leaves for electrolyte leakage (Figure S1). The conductivity of the *Bax* was always higher than that of each gene or complex overexpression, and *VpPR10.1*/*VpVDAC3* took the second place.

Cytochrome c is exclusively located in the mitochondria and serves as an electron shuttle signal inducing mitochondrial‐mediated apoptosis (Circu and Aw, 2010; Esmaeili *et al*., 2016; Lei *et al*., 2006), so we used antibody recognizing cytochrome c to examine the release after transient expressed with *VpPR10.1* or *VpVDAC3*. In *VpPR10.1* overexpressed leaves, antibody against cytochrome c signal appeared both in the total protein and in the mitochondrial enriched fraction. Meanwhile, in *VpVDAC3* overexpression and co‐expression samples, cytochrome c was also detected in the cytoplasm(Figure 5c). *Metacaspase* is an important component in cell death (Coll *et al*., 2010; Kim *et al*., 2003, 2006). As an additional means to assess cell death, we evaluated the expression of *NbMCA1* in *N. benthamiana* (Figure 5d). Compared to the GFP control, the co‐expression of VpPR10.1 and VpVDAC3 induced more than a fourfold induction after 48 h of agro‐infiltration.

### Generation and expression pattern analysis of *VpPR10.1* transgenic grapevine

**Figure 6:**
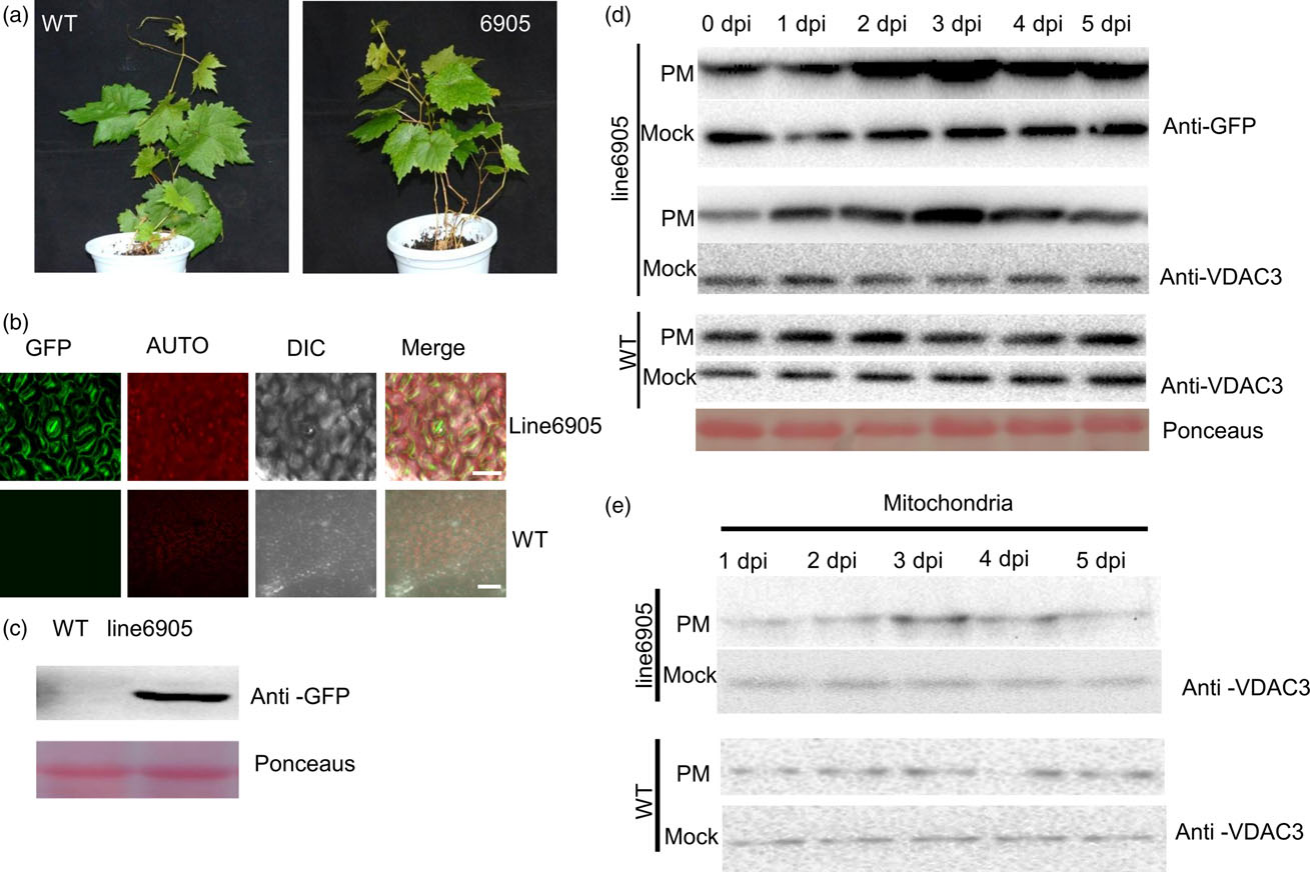
Characterization and Expression Pattern Analysis of *VpPR10.1* Transgenic Grapevine. (a) Phenotypic study of transgenic line 6905 and wild type. *VpPR10.1* transgenic line 6905 and WT grown in the feeding block for 40 days (left, ‘Thompson Seedless’, right, transgenic line 6905). Bars = 1 cm. (b) Direct GFP fluorescence observation of grape leaf abaxial surface from transgenic line 6905 and WT using confocal microscope. Bars = 50 μm. (c) Western blot showing expression levels of insert gene *VpPR10.1* with GFP tag in transgenic line 6905 and WT. Ponceaus indicates control loading. (d) Expression patterns of VpPR10.1 and VpVDAC3 in transgenic line 6905 under infection of *Plasmopara viticola*. PM,* P. viticola* induction; Mock, inoculated with double distilled water. Ponceaus indicates control loading. (e) Expression patterns of VpVDAC3 in enriched mitochondria from transgenic line 6905 under post of *P. viticola*. Protein was detected by Western blot using anti‐VDAC3 as described in the experiment and procedure section.

To explore function of *VpPR10.1* in grapevine, we overexpressed *VpPR10.1* in susceptible cultivar *V. vinifera* cv. ‘Thompson Seedless’, and under the normal grow conditions, features of transgenic grapevine line 6905 showed no significant differences with nontransgenic control (Figure 6a). We also used laser scanning confocal microscopy to check GFP fluorescence directly, in leaves of line 6905 and nontransgenic control (Figure 6b). Line 6905 showed strong GFP fluorescence located in whole cells including stoma. Proteins from transgenic grapevine line 6905 and WT were extracted to detect the antibody against GFP. Western blot showed clearly that the transgenic grapevine line 6905 exhibited expression of *VpPR0.1*‐GFP (Figure 6c).

GFP antibodies and the antibody against VpVDAC3 were used in immunoblot analysis of grapevine leaves inoculated with *P. viticola* (Figure 6d). The immunoblot results indicate that upon *P. viticola* inoculation, both VpPR10.1 and VpVDAC3 were accumulated in line 6905. To confirm the function of *VpVDAC3* under pathogen attack, we evaluated the expression of *VpVDAC3* in extracted mitochondrial proteins (Figure 6e). Compared to no obvious change in mock, VpVDAC3 also accumulated in the mitochondrial extracted from transgenic grapevine line 6905 inoculated with *P. viticola*.

### VpPR10.1‐GFP transgenic grapevine line show enhanced resistance against *P. viticola*

**Figure 7:**
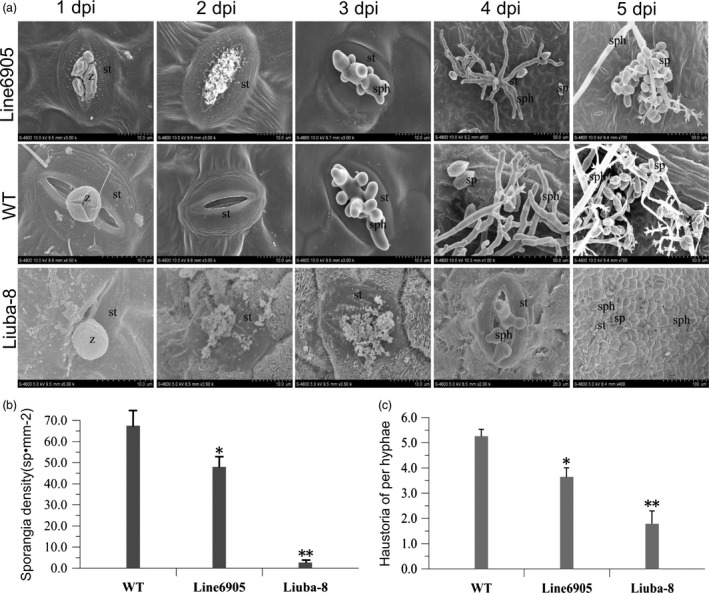
Overexpression of *VpPR10.1* limits the development of *Plasmopara viticola*. The sporangia of *P. viticola* were attached to the back of leaf discs surface from WT and the overexpressing VpPR10.1 line 6905. *P. viticola* highly resistant *Vitis piasezkii* ‘Liuba‐8’ was used as positive control. (a) Scanning electron microscopy observation of *VpPR10.1* transgenic line 6905 and WT leaves inoculated with *P. viticola* from 1 to 5 dpi. Images represented three independent experiments, z, zoospores encystment; st, stomata; sp, sporangia; sph, sporangiophores; hy, hyphae. (b) Average sporangia density of *P. viticola* from different genotypes at 5 dpi. Error bars indicate SE (*n* = 3) from three independent biological replicates (*P <* *0.05 and **P <* *0.01, *t*‐test). (c) Number of haustoria per hyphae on a total of 20 infected discs from different grapes at 48 hpi. Error bars indicate SE from three independent biological replicates (*P <* *0.05 and **P <* *0.01, *t*‐test).

*Plasmopara viticola* development in transgenic grapevine line and nontransgenic control was visualized by scanning electron microscopy (Liu *et al*., 2015). In *VpPR10.1* transgenic line 6905, stomatal blockage was appeared in leaf discs as early as 2 dpi (Figure 7a). This is a resistance characteristic consistent with previous observation (Gindro *et al*., 2003; Trouvelot *et al*., 2008). By 3 dpi, differences in hyphal and sporangiophore growth between transgenic line 6905 and WT were observed (Figure 7a). Hyphae, primary hyphae and sporangia were detected in both *VpPR10.1* transgenic line 6905 and WT at 4 dpi. At five days after inoculation, the sporangia were shed from the sporangiophores of both genotype lines. Development of *P. viticola* in the highly resistant *V. piasezkii* ‘Liuba‐8’ was obviously inhibited (Figure 7a), while the suppression of *P. viticola* in the transgenic line 6905 was also apparent. Compared with Liuba‐8 and WT (Figure 7b), sporangia density in ‘Liuba‐8’ (1.8) was lower than that in the transgenic line 6905 (45.2), in susceptible WT the sporangia density was 67.4/mm^2^ (Figure 7b). The number of haustoria per hyphae at 48 hpi in the transgenic line 6905 (3.5) was lower than that in susceptible grape WT (5.2), but still higher than that in Liuba‐8 (1.6) (Figure 7c).

**Figure 8:**
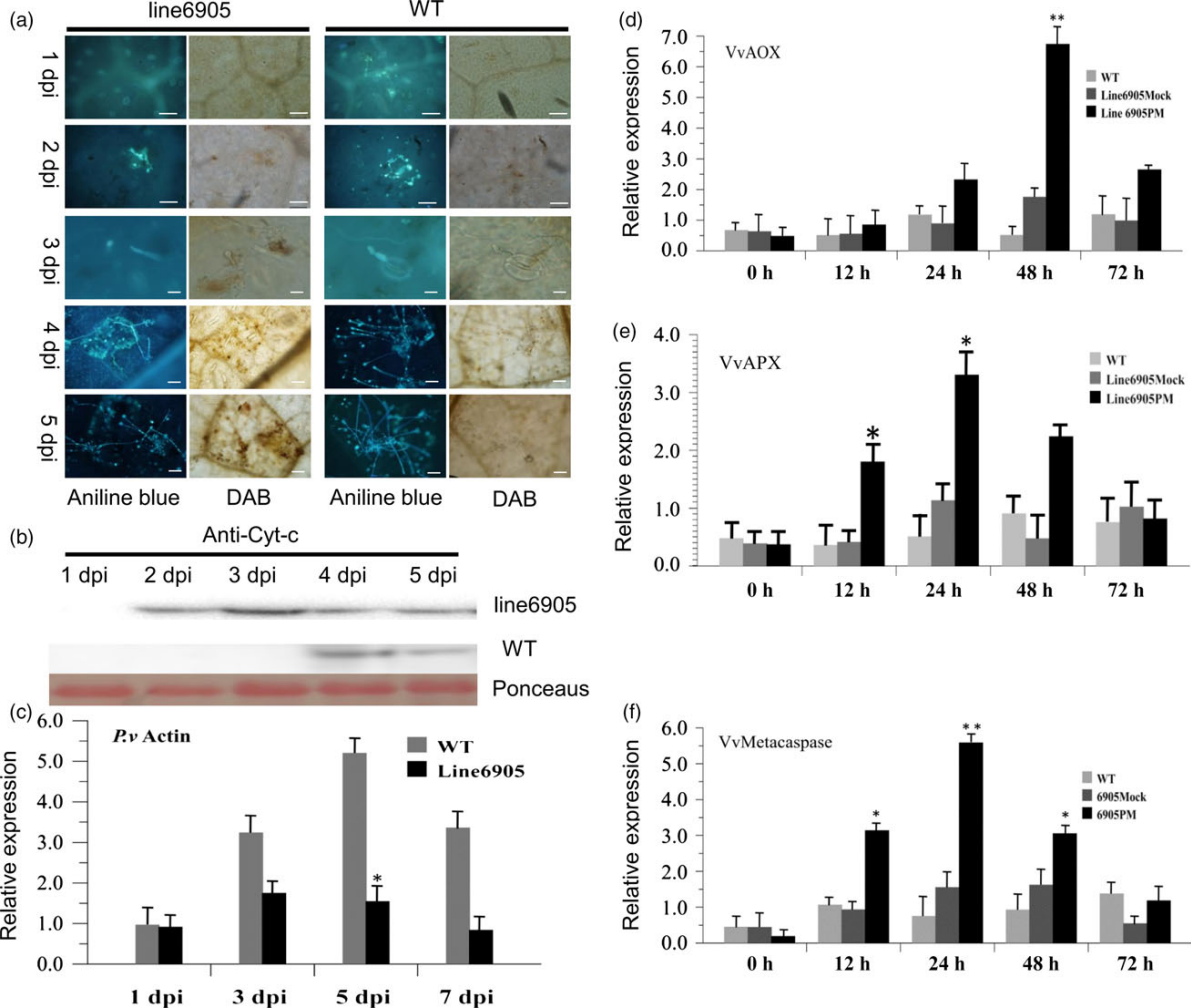
*VpPR10.1* overexpression enhances H_2_O_2_ accumulation during infection. (a) Fluorescence micrographs of oomycete development were stained with aniline blue (left); H_2_O_2_ accumulation was stained with DAB. Bar = 50 μm. (b) Western blot for detecting the cytochrome c in cytoplasm after inoculation with *Plasmopara viticola* for 1–5 dpi in transgenic line 6905; ponceaus indicates control loading. (c) RT‐PCR was carried out to evaluate the *actin* gene of *P. viticola* in VpPR10.1 transgenic line 6905 and wild‐type *Vitis vinifera* ‘Thompson Seedless’. *Vitis* 18s rRNA was used as reference gene (*P < 0.05) (*t*‐test). (d, e) The expression level of ROS‐related gene *VvAOX*,* VvAPX* in transgenic line 6905 and WT after inoculation with *P. viticola* at indicate times. Each value is the mean ± SE of three independent biological determinations. Asterisks indicate significant difference from mock control, *P < 0.05; **P < 0.01 (*t*‐test). (f) The expression level of apoptosis‐related gene (type II metacaspase) *Vvmetacaspase5* (Zhang *et al*., 2013) in transgenic line 6905 and WT after inoculation with *P. viticola* at indicated times. Asterisks indicate significant difference from the mock control, *P < 0.05, **P < 0.01(*t*‐test).

To determine whether the observed *VpPR10.1*‐mediated resistance to *P. viticola* was related to ROS accumulation, H_2_O_2_ production was evaluated using DAB staining. In VpPR10.1 transgenic line 6905, distinct DAB deposits appeared at 3 dpi (Figure 8a). By 4 dpi, altered hyphae could be seen in the most strongly stained zones. *VpPR10.1* overexpression grapevine line 6905 showed enhanced ROS production after *P. viticola* inoculation. These results suggest in *VpPR10.1* transgenic line, *P. viticola* development was restricted by ROS accumulation. At the same time, cytochrome c was detected in the cytoplasm as early as 2 dpi in line 6905, while in nontransgenic grape, it was detected after 4 dpi (Figure 8b). Taken together, these results suggest that *VpPR10.1* and *VpVDAC3* responded to the invasion of *P. viticola*. We also recorded the development of *actin* of *P. viticola* (Schmidlin *et al*., 2008) by RT‐PCR, and *P. viticola* in the *VpPR10.1* transgenic line 6905 was repressed compared to WT (Figure 8c).We also evaluated the expression of ROS‐related genes by RT‐PCR, as shown in Figure 8d, in the *VpPR10.1* transgenic line, inoculation with *P. viticola* induced 14‐fold higher expression of *VvAOX* compared to the WT(Figure 8d), and at the same time, the transcriptional level of *VvAPX* was also increased (Figure 8e). Expression of *Vvmetacaspase5* was also strongly induced by *P. viticola* in line 6905 (Figure 8f), we overexpressed *Vvmetacaspase*4,5,6 fused with GFP in *N. benthamiana*, and each resulted in a different level of ROS accumulation and cell death (Figure S2). From these results, we infer the overexpression of *VpPR10.1* in susceptible grapevines increased the original resistance to *P. viticola*.

## Discussion

Cultivated grape originates from the European grape (*Vitis vinifera*), the disease resistance properties available within *V. vinifera* are limited, and their disease susceptibility is not really compatible with its dominant position as major fruit crop. Great harm from downy mildew caused by *P. viticola* seriously affects both the yield and the quality of grapes (Brewer and Milgroom, 2010). In this study, we found that the *VpPR10.1* transgenic line showed increased resistance to *P. viticola* (Figures 7 and 8). In previous research, VpPR10.1 inhibited the development of ascomycete *Alternaria alternate*, and when recombinant VpPR10.1 or the mutations are incubated with tobacco BY2s suspension cells, resulting in degrees of PCD and DNA degradation (Xu *et al*., 2014), it is possible that *VpPR10.1* mediates antifungal activity in grape host cells.

PR10 contributes to resistance in pepper through the interaction with LRR1, and the cytoplasmic localization of PR10/LRR1 is responsible for the induction of cell death (Choi *et al*., 2012). In our research, VpPR10.1 together with mitochondrial located VDAC (Figure 2) enables defence responses in grapevine (Figures 7 and 8). PR10s may participate in different pathways to regulate resistance. Decoding the activation of defence responses is key step to understanding how plant immunity systems work (Postma *et al*., 2016). Reactive oxygen species are important transducers of induced immune responses even cell death (Jaleel *et al*., 2009; Miller *et al*., 2010; Van Breusegem *et al*., 2001). In our study, VpPR10.1 positively regulates VpVDAC3‐mediated cell death along with the generation of ROS (Figures 3 and 5). PR10 may take part in the defence responses linked with cell death.

The subcellular location *VpVDAC3* in *N. benthamiana* protoplasts suggests that it is located in mitochondria (Figure 2), which function in transporting through the inner and outer membranes (Colombini, 2004;) . Predicatively located in the outer membrane of mitochondria, *VDAC* takes part in the transfer of molecules and macromolecules through the mitochondrial membranes (Benz, 1994; Colombini, 1979). Evidences suggest that VDAC is a unique protein participating in mitochondria‐mediated cell death (Abu‐Hamad *et al*., 2006; Schein *et al*., 1976). Studies also show clearly that apoptosis induced by VDAC exists in yeast, rice, fish and murine (Ghosh *et al*., 2007; Godbole *et al*., 2003; Zaid *et al*., 2005). We found that overexpression of *VpVDAC3* in *N. benthamiana* triggered cell death (Figure 5a), and that *metacaspase*‐like gene was induced expression (Figure 5d). Plant *metacaspase* plays important roles in PCD, and the underlying mechanisms remain obscure (Cambra *et al*., 2010; Hoeberichts *et al*., 2003).

Voltage‐dependent anion channel is important in receiving signals and activating the cell death‐like responses, and release of cytochrome c in mitochondria is one of the key steps in apoptosis (Pinto *et al*., 2010). Previous study showed that hexokinase promotes voltage‐dependent anion channel closure and prevents mitochondria‐mediated apoptotic cell death (Azoulay‐Zohar *et al*., 2004), VIGS of mitochondria‐associated hexokinase *Hxk1* of *N. benthamiana* resulted in PCD and cytochrome c release, and caspase‐9‐like and caspase‐3‐like proteolytic showed strongly activities (Kim *et al*., 2006). Our research shows the co‐expression of *VpPR10.1* and *VpVDAC3* in *N. benthamiana* also results in the release of cytochrome c (Figure 5c).

Many earlier studies focused on the overexpression of *VDAC* in plant disease resistance as well as abiotic stress (Al Bitar *et al*., 2003; Desai *et al*., 2006; Lee *et al*., 2009; Tateda *et al*., 2009; Wandrey *et al*., 2004; Yang *et al*., 2011; Zhang *et al*., 2015). Here, we find that VpPR10.1 interacts with VpVDAC3 (Figure 1), and VpVDAC3 was induced by *P. viticola* in grapevine, the results enriched the functional research of *VDAC*. In previous studies, *VDAC* has been shown to enhance mitochondria‐triggered cell death in *Arabidopsis* and tobacco (Shoshan‐Barmatz *et al*., 2010). In this study, we confirm the interactions between VpPR10.1 and VpVDAC3 to activate defence responses in grape. In contrast with previous findings, *VpPR10.1* enhances *VpVDAC3* triggered resistance as a positive modulator in grape. The transient co‐expression of *VpVDAC3* with *VpPR10.1* intensifies the cell death response (Figure 5a). Although showed no apparent phenotype, transgenic grapevine line 6905 exhibited a higher resistance to *P. viticola* (Figure 6, 7 and 8). The transgenic line of *VpPR10.1* also presents disease‐associated cell death upon inoculation with *P. viticola*, which is accompanied by ROS generation and cytochrome c release (Figure 8a and 8b).

During plant‐pathogen interactions, the production of ROS plays a critical role in HR‐like defence responses (Torres *et al*., 2006). The most successful ROS studied are NADPH oxidases. As one of NADPH‐related genes in plants, *Rbohs* plays important roles in plant stress responses (Kaur *et al*., 2014). Besides NADPH, mitochondria also provide complementary sources of ROS and the reaction to oxidative stress (Xie and Chen, 1999).The mitochondrial pathway functions together with the generation of ROS lead to oxidative damage to whole cell (Song *et al*., 2009).There are other features of ROS, for example H_2_O_2_, which directly kills pathogen, inducing plant toxins (Doke, 1983), causing hypersensitive response (Delledonne *et al*., 2001) and operating resistance (Dat *et al*., 1998).

Many studies show overmuch ROS result in cell death may act as a defence role involving in plant stress responses (Torres *et al*., 2006). For pathology, there is much evidence that ROS act as signal transducers (Chen *et al*., 1993). Even so, the route of signal transduction by ROS still remains unclear. Vanacker *et al*. (2000) found that in disease resistant leaves, areas infected with hyphae suffer rapid accumulations of H_2_O_2_ and the formation of mastoid deposits, which are not found in susceptible varieties.

Voltage‐dependent anion channels involved in H_2_O_2_ accumulation has been confirmed, and during pathogen infections, H_2_O_2_ was accumulated in *N. benthamiana* overexpressed VDAC and was reduced in *N. benthamiana* lack VDAC (Tateda *et al*., 2009). AtVDAC3 from *A. thaliana* was shown to be involved in ROS generation (Zhang *et al*., 2015). Nevertheless, the mechanism of ROS production via VDACs is undefined, in our attempt to confirm the possible ROS induction of VpVDAC3, we transiently expressed *VpVDAC3* in *N. benthamiana*. The overexpression of *VpVDAC3* in *N. benthamiana* caused ROS accumulation and increased related gene expression thus leading to cell death (Figures 3, 5 and 8). Furthermore, when we exposed *P. viticola* to *VpPR10.1* transgenic grapes, ROS were generated (Figure 8a) and *VpPR10.1* and *VpVDAC3* mediate the resistance to *P. viticola*, which involved in the burst of ROS together with cytochrome c release (Figure 8b).

VDAC oligomeric structure is still not clear, and the N‐terminal oligomerization of VDAC is greatly enhanced upon apoptosis and directly associated with cytochrome c release (Keinan *et al*., 2010; Shoshan‐Barmatz *et al*., 2010). In this research, we tested the interaction between different truncated VpVDAC3 fragments and VpPR10.1, and the result indicated that N‐terminal and C‐terminal VpVDAC3 showed interaction with VpPR10.1 (Figure S3). As N‐terminus is the oligomeric site of VDAC in animal, we suggest that VpPR10.1 may have the same function to promote the ability of VpVDAC to enhance the opening of MPTP, finally induce cytochrome c release and subsequent cell death.

VDACs may affect cellular redox metabolism by producing metabolites, but the precise mechanisms of VDACs in fungal resistance remain unclear (Homblé *et al*., 2012; Kusano *et al*., 2009; Takahashi and Tateda, 2013; Tikunov *et al*., 2010; Zhang *et al*., 2015). Many studies focus on the cell death induced by cytochrome c release and caspase activation (Kluck *et al*., 1997; Li *et al*., 1997; Liu *et al*., 1996; Yang *et al*., 1997), and the release of cytochrome c is especially responsible for the mitochondria‐mediated apoptotic pathway. We successfully identified the interaction between VpPR10.1 and VpVDAC3 (Figure 1). The overexpression of *VpPR10.1* from resistant grape *V. pseudoreticulata* ‘Baihe‐35‐1’ in WT (nontransgenic Thompson Seedless) enhanced the resistance of the transgenic plant by causing ROS accumulations following downy mildews infection (Figure 8). Combining the above findings, we propose a model for the synergistic action of VpPR10.1 and VpVDAC3 in grapevine (Figure S4). Once pathogen infection occurs, pathogen‐related proteins of the host cell along with VpVDAC3 induce defence reactions, resulting in release of cytochrome c via the *VDAC* channel and the accumulation of ROS. Nevertheless, the accumulation of ROS may cause oxidative damage to mitochondrial and cellular proteins and the cell death‐like resistance response finally came up. Together these are responsible for destroying the infected cell, as well as promoting the HR‐like cell death and the defence action. These findings facilitate molecular breeding efforts to improve downy mildew resistance in grapevine.

In the previous study, the pepper pathogenesis‐related protein 10 (PR10) was identified as a leucine‐rich repeat protein 1 (LRR1) interacting partner, and the LRR1/PR10 complex formed in the cytoplasm is responsible for cell death‐mediated defence (Choi *et al*., 2012). In this study, we identify VpVDAC3 as interaction partner of VpPR10.1 to regulate defence response to *P. viticola* (Figure 5 and 8). The VpPR10.1/VpVDAC3 complex is also responsible for cell death. These findings come up with the possibility that PR10.1 may be involved in cell death‐related immunity through forming different complexes with different pathways in plants. These results enrich our understanding of the function of PR10. However, much more detailed study is needed for the molecular mechanisms underlying how VpPR10.1 biochemically affects VpVDAC3 in the future.

## Experimental procedures

### Plant materials and growth conditions

Grapevine and *N. benthamiana* were planted in an illuminated incubator with a day/night cycle of 25 °C 14‐h light/20 °C 10‐h dark. The transformation of grapevine was referred to the description previously published (Zhou *et al*., 2014).

### Yeast two‐hybrid assay

The Y_2_H Gold system was conducted under instructions of the manufacturer's (Clontech, Dalian, China, http://www.clontech.com/. The *P. viticola* induced prey cDNA library of *V. piasezkii* ‘Liuba‐8’ that one of Chinese wild grapevine was constructed according to the user's manual of Make Your Own ‘Mate & Plate TM’ Library System (Clontech). *VpVDAC3* and *VpPR10.1* were cloned and recombined into the pGADT7 and pGBKT7 to constructed BD/*VpPR10.1*, AD/*VpVDAC3*, BD/*VpVDAC3* and AD/*VpPR10.1*. Logically, the conjugated constructs were transformed into Y_2_H Gold according to the Yeastmaker™ Yeast Transformation System 2 User Manual. The positive strains were selected on SD/‐Leu/‐Trp/‐Ade/‐His medium containing 50 μg/mL X‐α‐Gal and 150 ng/mL aureobasidin A (AbA). Combinations of (AD/T) with BD/p53 and BD/Lam were, respectively, served as positive and negative controls.

### BIFC analysis

For the BIFC constructs, both the coding regions of genes were cloned into the plasmid pUC‐SPYNE to build *VPR10.1*‐YFPNE and *VpVDAC3*‐YFPNE and into pUC‐SPYCE to form *VPR10.1*‐YFPCE and *VpVDAC3*‐YFPCE, resulting in pSPYNE‐*VpPR10.1*,* VpVDAC3‐*pSPYCE and *vice versa* as described by Walter (Walter *et al*., 2004). Corresponding BIFC plasmids and negative controls were co‐expressed in *N. benthamiana* protoplasts. Twenty hours after transformation, the protoplasts were visualized and captured using an Olympus FV1000 LCS confocal laser scanning microscope (Olympus).

### Co‐IP

For CO‐IP, *VpPR10.1* and *VpVDAC3* were cloned into p35S, GFP (*VpPR10.1*‐GFP) and p35S, 3Flag (3Flag‐*VpVDAC3*) vector. *Agrobacterium* strain GV3101 containing the constructs was infiltrated into *N. benthamiana* leaves. 0.7 g flash‐frozen homogenized *N. benthamiana* leaf tissue was suspended in 1.5 mL IP buffer (Choi *et al*., 2012), followed by incubation with monoclonal anti‐GFP (ABclonal) overnight at 4 °C with gentle shaking. The immune complexes were incubated with prewashed monoclonal anti‐GFP agarose (Sigma‐Aldrich) for 5–6 h at 4 °C and were centrifuged and washed with immunoprecipitation buffer (Choi *et al*., 2012). Resuspend proteins were separated by 10% SDS‐PAGE. Anti‐GFP mouse monoclonal antibody (ABclonal) and flag‐specific monoclonal antibodies (Santa Cruz) were both probed at 1,2000 dilution to detect goal proteins. IPKine™ HRP Goat Anti‐Mouse IgG HCS (http,//www.abbkine.com/) was used as a secondary antibody.

### Pull‐down

Recombinant proteins GST‐*Vp*PR10.1 and MBP‐*VpVDAC3* were purified with glutathione resin (Novagen, Germany) and amylose resin (NEB, England) according to methods described previously (Xu *et al*., 2014). Proteins GST‐*Vp*PR10.1 and MBP‐*VpVDAC3* were individually dialysed in protein dialysis buffer [50 mm Tris‐HCl (pH7.4), 200 mm NaCl, 1 mm EDTA, 1 mm DTT, 50% glycerol], and purified bait protein MBP‐VpVDAC3 (MBP as control) was added to 25 μL amylose resin (beads) and incubated in ice for 1 h. Then, prey protein GST‐*Vp*PR10.1 (GST as control) was loaded onto the beads. After the pull‐down assay for 5 h at 4 °C, the beads were precipitated and washed with buffer [50 mm Tris‐HCl (pH7.4), 200 mm NaCl, 1 mm EDTA, 0.05% Nonidet P‐40]. Proteins detained on the beads were released and denatured by boiling the bead pellet with 5 × SDS‐PAGE loading buffer, then detected by GST and MBP antibody, respectively.

### *Plasmopara viticola* infection and scanning electron microscopy observation

*Plasmopara viticola* was prepared as a sporangial suspension. The inoculation was according to the description of Liu (Liu *et al*., 2015), and leaf pieces were collected from 1 dpi to 5 dpi and immediately fixed with 4% (v/v) glutaraldehyde in 0.2 m pH 6.8 phosphate buffer saline. After this, they were dehydrated by aqueous ethanol series (30%, 50%, 70%, 80%, 90% and 100%) for 30 min each. The samples were observed with Hitachi S‐4800 FE‐SEM. To evaluate the susceptibility, the number of haustorias of each hypha in three different genotype was quantified at 48 hpi (hours postinoculation) and the sporangia density of each mm^2^ was counted and calculated at 5 dpi.

### Histochemical staining and microscopy

DAB staining was carried out as previously described (Daudi and O'Brien, 2012). Aniline blue staining was performed according to Liu *et al*. (2015), and Olympus BX‐51 was used for microscopic observation of the *P. viticola* in grapevine.

### *Agrobacterium*‐mediated transient expression

*VpPR10.1* and *VpVDAC3* were cloned into pCambia2300‐GFP and 3Flag ‐pCambia1307, respectively. *Agrobacterium* strain GV3101 carrying the constructs were infiltrated into fully expanded *N. benthamiana* leaves, and *Bax* was also transiently expressed as comparison (Du and Hwang, 2011; Lacomme and Santa Cruz, 1999).

### Protein extraction and western blot

The infiltrated leaves were immediately harvested in liquid nitrogen and ground to homogeneous powder. Equal volumes of PPEB buffer (1.0 m Tris‐HCl, pH 8.0, 10% SDS, 50% glycerol, 5% mercaptoethanol) were used to extract the denatured proteins in leaves of *N. benthamiana* or grapevine. 700–800 mg of leaf powder was used. Followed boiling for 10 min, samples were centrifuged at 4 °C for 20 min at 14 000 g. The BCA Protein Assay Kit (CWbiotech, Beijing, China, CW0014) was used to quantify the protein. The supernatant was mixed with 5 ×  SDS buffer and then re‐boiled for 5 min followed fractionation before 10% SDS‐PAGE. Images were captured using ChemiDoc™ XRS+ Software. For the preparation of VpVDAC3 antibody, PET32a‐*VpVDAC3* construct was transformed into *E. coli* BL21 strain for protein induction. The purified fusion protein was used to immune a New Zealand white rabbit to produce antibody against VpVDAC3 protein in Proteintech Group, Inc.

### Grapevine transformation

*VpPR10.1* was constructed into the pCambia2300‐GFP vector and was transformed into *Agrobacterium* strain GV3101. Then, *VpPR10.1* was transformed into somatic embryos of *V. vinifera*. cv. ‘Thompson Seedless’ system established previously (Zhou *et al*., 2014).

### *Nicotiana benthamiana* protoplast separation and gene localization

Protoplasts were isolated from healthy, nonsenescent, fully expanded leaves of *N. benthamiana* as described by Yoo *et al*. (2007). For the subcellular localization assay, fluorescence was visualized using an FV1000MPE Olympus laser confocal microscope.

### Mitochondrial fractionation

The mitochondrial fractions were processed following the instructions of Plant Mitochondria Purification Kit (TIANDZ, Hangzhou, China), 4.5 g fresh *N. benthamiana* leaves was used, and mitochondria were collected in the precipitate. Finally, the mitochondria were re‐suspended in mitochondrial store buffer and stored in a −80 °C ultra‐low temperature freezer.

### Detection of reactive oxygen species and mitochondria depolarization

Hydrogen peroxide was stained with DAB, and ROS assay kits (Beyotime) were used to determine the ROS levels. After infiltration of individual genes for 60 h in *N. benthamiana*, protoplasts were isolated. After incubated with DCFH‐DA at 37 °C for 20 min at a concentration of 2 μm, protoplast samples pretreated with DPI act as control. For JC‐1 dye, protoplasts were incubated with 10 mg/mL JC‐1 for 30 mins at 25 °C. The fluorescence of DCF and JC‐1 was visualized and captured using a Carl Zeiss LSM 510 confocal, and 20 *N. benthamiana* protoplasts were captured.

### RT‐PCR analysis

RNA was extracted using the plant RNA kit (OMEGA R6827‐01). For RT‐PCR, the SYBR Premix ExTM TaqII kit (Takara, Dalian,China, http//www.takara.com.cn/ was used, and RT‐PCR was carried out in an iCycler iQ 5 thermal cycler (Bio‐Rad), using a two‐step protocol described by Han *et al*. (2016). *Pv*‐actin (He *et al*., 2013) was evaluated by RT‐PCR between WT and *VpPR10.1* transgenic line 6905 after inoculation with *P. viticola*. Grapevine 18s rRNA was used as internal reference (*P < 0.05, *t*‐test).

## Supporting information


**Figure S1** Quantification of Conductivity in Different Genes.
**Figure S2** Transient Expression of *Vvmetacaspases* in *Nicotiana benthamiana* induce ROS accumulation and cell death. Phenotypic and physiological analyses of following (A)Upper two lines, DAB stain of *Bax, Vvmetacaspase4, 5, 6* and *GFP* were all injected in one *N. Benthamiana* leave in 1 dpi, 3 dpi, 5 dpi and 7 dpi. The bluish colour of DAB staining represents the accumulated H_2_O_2_. Bottom two lines, trypan blue stain of *Bax, Vvmetacaspase4, 5, 6* and *GFP* injected in one *N. Benthamiana* leave. (B) Checking of *Vvmetacaspase4, 5, 6* and *GFP* protein by Western blotting in *Agro*‐infiltration(od = 0.75) *N. benthamiana* leaves. Ponceaus staining stand for control loading.
**Figure S3** Truncate VpVDAC3 Interaction with VpPR10.1.
**Figure S4** Proposed Model of *VpPR10.1* Mediated Resistance Response by Interaction with *VpVDAC3*.
**Table S1** The summary of positive clones obtained from Chinese wild grape *V. piasezkii* Liuba‐8 after *P. viticola* infection cDNA library using VpPR10.1 as bait.
