# Supplementary material for: Grapevine VpPR10.1 functions in resistance to Plasmopara viticola through triggering a cell death‐like defence response by interacting with VpVDAC3
Source: Plant Biotechnol J. 2018 Mar 8;16(8):1488–501. doi: 10.1111/pbi.12891 (PMC6041444; doi:10.1111/pbi.12891)
Supplement: Supplementary file 1 — Figure S1 Quantification of Conductivity in Different Genes. Figure S2 Transient Expression of Vvmetacaspases in Nicotiana benthamiana induce ROS accumulation and cell death. Phenotypic and physiological analyses of following (A)Upper two lines, DAB stain of Bax, Vvmetacaspase4, 5, 6 and GFP were all injected in one N. Benthamiana leave in 1 dpi, 3 dpi, 5 dpi and 7 dpi. The bluish colour of DAB staining represents the accumulated H2O2. Bottom two lines, trypan blue stain of Bax, Vvmetacaspase4, 5, 6 and GFP injected in one N. Benthamiana leave. (B) Checking of Vvmetacaspase4, 5, 6 and GFP protein by Western blotting in Agro‐infiltration(od = 0.75) N. benthamiana leaves. Ponceaus staining stand for control loading. Figure S3 Truncate VpVDAC3 Interaction with VpPR10.1. Figure S4 Proposed Model of VpPR10.1 Mediated Resistance Response by Interaction with VpVDAC3. Table S1 The summary of positive clones obtained from Chinese wild grape V. piasezkii Liuba‐8 after P. viticola infection cDNA library using VpPR10.1 as bait. [file PBI-16-1488-s001.docx]

**
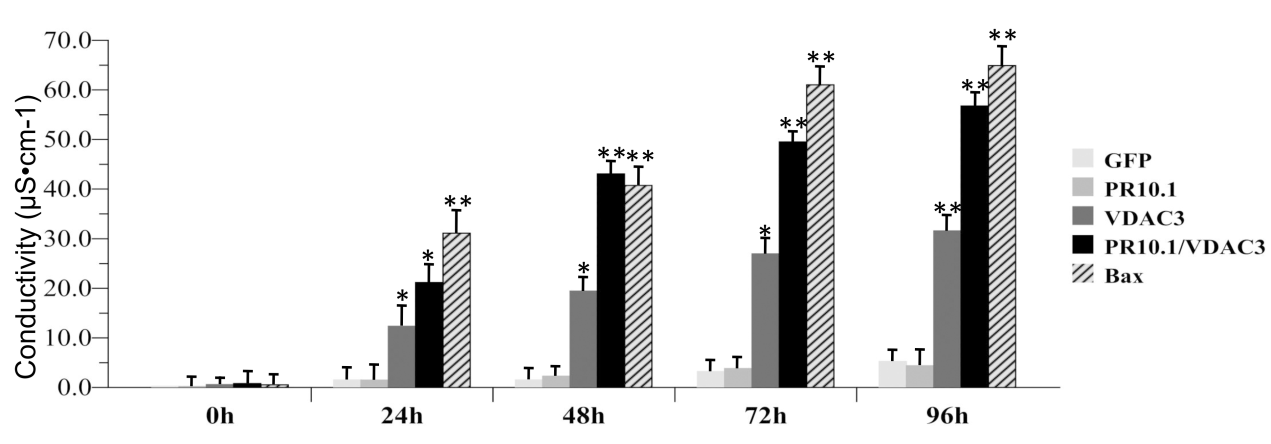
Supplemental Fig. 1** **Quantification of Conductivity in Different Genes**

Quantification of electrolyte leakage from infiltrated leaf discs. After transient expression with GFP, VpPR10.1, VpVDAC3, VpPR10.1/VpVDAC3, BAX. *Nicotiana benthamiana* leaf discs (1 cm in diameter) from each gene were used to measure ion leakage (Lee et al., 2011). Asterisks indicate significant difference between each gene . and GFP control, *P <0.05; **P < 0.01.

***
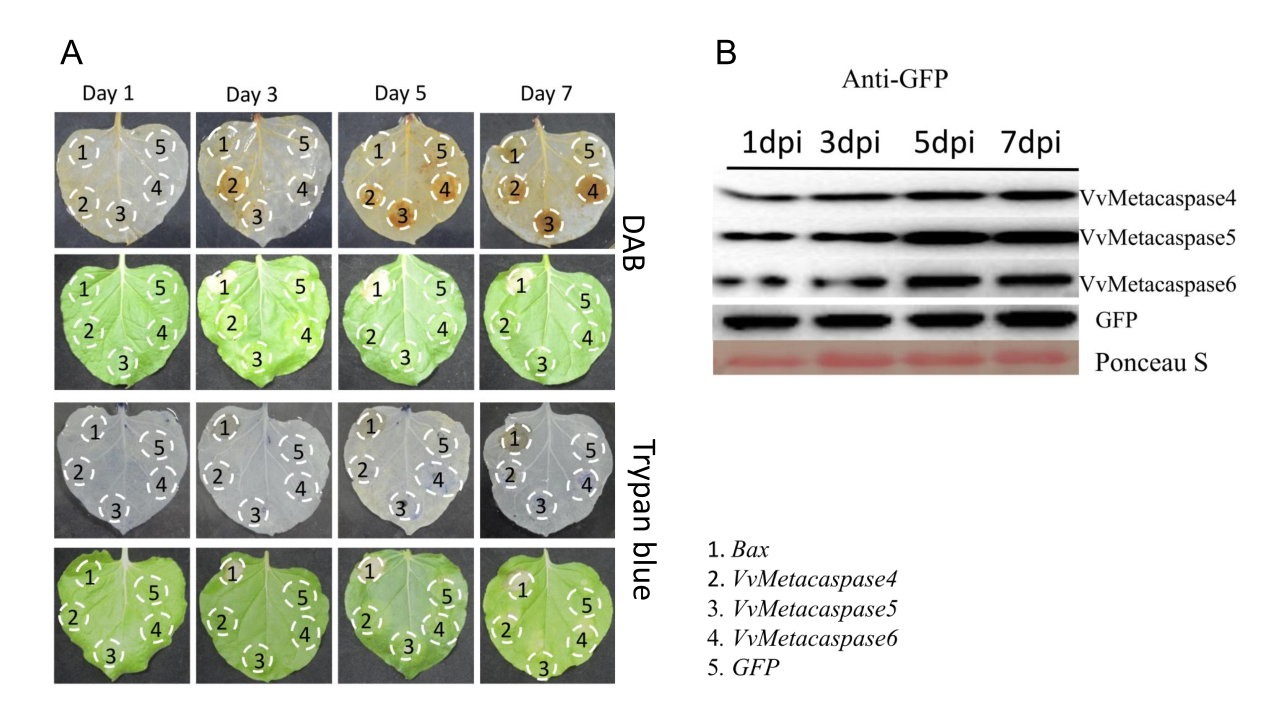
***

**Supplemental Fig. 2. Transient Expression of *Vvmetacaspases* in *N. benthamiana* induce ROS accumulation and cell death.**

Phenotypic and physiological analyses of following (A)Upper two lines, DAB stain of *Bax,* *Vvmetacaspase4, 5, 6* and *GFP* were all injected in one *N. Benthamiana* leave in 1 dpi, 3 dpi, 5 dpi and 7 dpi. The bluish color of DAB staining represents the accumulated H_2_O_2_. Bottom two lines, trypan blue stain of *Bax,* *Vvmetacaspase4, 5, 6* and *GFP* injected in one *N. Benthamiana* leave. (B) Checking of *Vvmetacaspase4, 5, 6* and *GFP* protein by western blotting in *Agro*-infiltration(od=0.75) *N. benthamiana* leaves. Ponceaus staining stand for control loading.


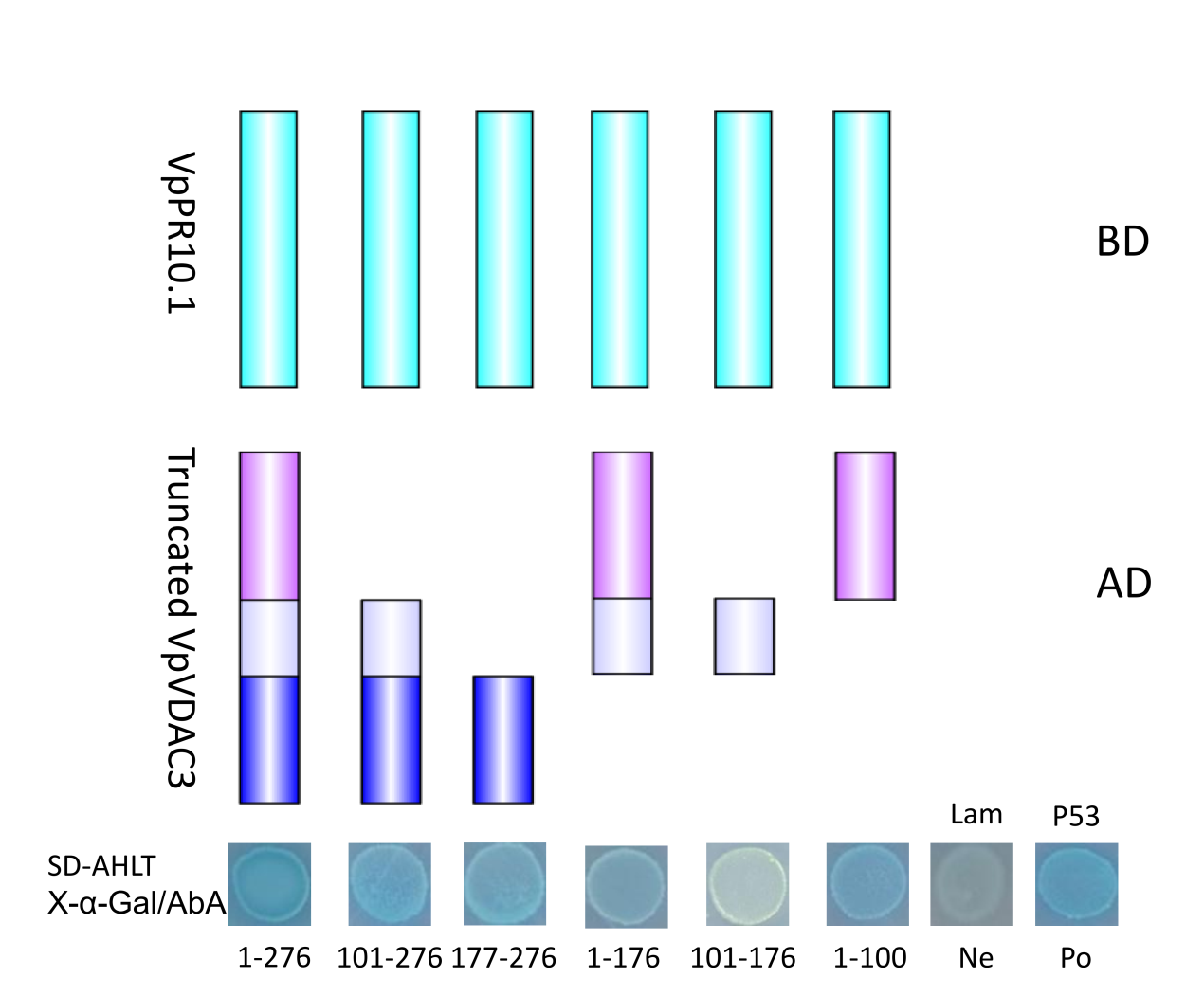


**Supplemental Fig. 3. Truncate VpVDAC3 Interaction with VpPR10.1**

Yeast two-hybrid assay. pGBKT7 or pGADT7 plasmid containing *VpPR10.1* and *VpVDAC3* 6 (Full length 1-276,1-100,1-176,101-176,101-276,176-276)oligopeptides were transformed into Y2H Gold. Combinations of the (AD/T) with BD/p53 and BD/Lam were used as positive and negative controls.


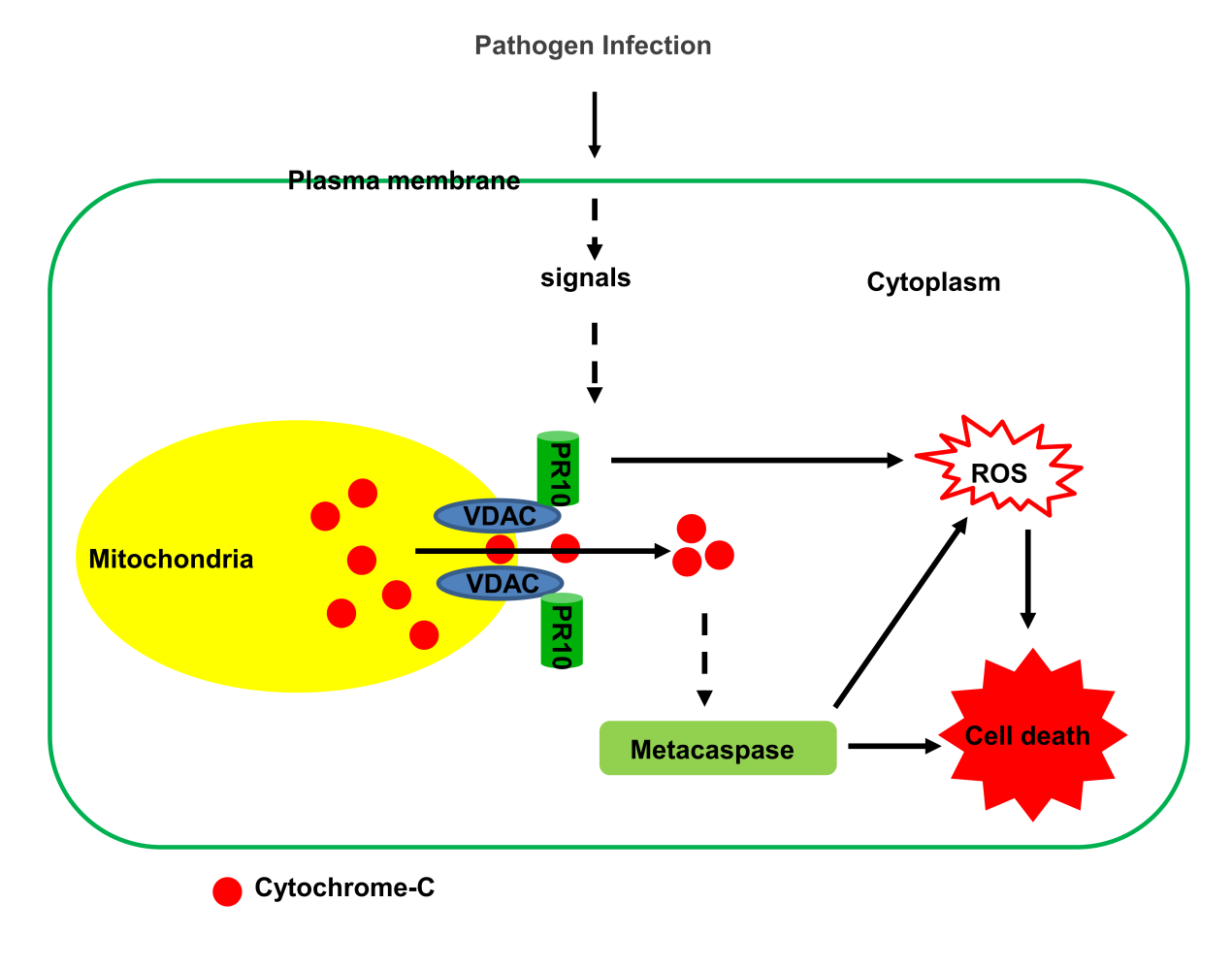


**Supplemental Fig. 4. Proposed Model of *VpPR10.1* Mediated Resistance Response by Interaction with *VpVDAC3***

| Accession number  **Table S 1.** The summary of positive clones obtained from Chinese wild grape *V. piasezkii* Liuba-8 after *P. viticola* infection cDNA library using VpPR10.1 as bait | Protein name | **Clone** number | **D**escription |
| --- | --- | --- | --- |
| *Vitis vinifera* polyphenol oxidase,1 (PPO1) | XP_002275842.2 | 2 | A copper-binding enzyme of the plant secondary metabolismthat can oxidize polyphenols to quinones |
| *Vitis vinifera* F-box protein At5g39450-like (F-box) | XM_002275467.1 | 1 | Functioning in numerous cellular mechanisms during plant developmental and physiological processes |
| *Vitis vinifera* DEAD-box ATPdependent RNA helicase 56 (DEAD-box) | XP_002268833.1 | 2 | Various biochemical activities involved in RNA-medicated processes and cellular processes including splicing, ribosome, biogenesis |
| *Vitis vinifera* cell number regulator 8-like (CNR8) | XP_002279879.1 | 3 | Functioning as cell number and size regulators |
| *Vitis vinifera* coatomer subunitbeta’ 2 (b’-COP) | XP_002272269.1 | 1 | An enzyme is essential and required for ER-to-Golgi transport |
| Vitis pseudoreticulata glyoxal oxidase (GLOX) | ACV49899.1 | 3 | A distinct binding pattern to proteins associated with producing H2O2 |
| *Vitis vinifera* receptor-like protein kinase2 | XP_010650225.1 | 2 | A novel factor controlling anther development |
| *Vitis vinifera* psbP domain-containing protein | XP_002265695.1 | 3 | A nuclear-encoded thylakoid lumenal protein, is essential for photosystem I assembly |
| *Vitis vinifera* uncharacterized protein | XP_010654864.1 | 12 | Uncharacterized protein |
| *Vitis vinifera* uncharacterized protein | XP_010646603.1 | 8 | Uncharacterized protein |
| *Vitis vinifera* uncharacterized protein | NP_001267959.1 | 5 | Uncharacterized protein |
| *Vitis vinifera* uncharacterized protein | XP_010649650.1 | 7 | Uncharacterized protein |
| *Vitis vinifera* zinc finger protein CONSTANS-like | XP_003635069.1 | 1 | Widely expressed in Eukaryotes, Participating  in cell differentiation, proliferation and apoptosis |
| *Vitis vinifera* mitochondrial outer membrane protein porin 2 | XP_002279650.2 | 4 | The most abundant protein of the outer membrane of mitochondria |
| *Vitis vinifera glyceraldehyde-3- phosphate dehydrogenase A (GAPDH)* | XP_002278352.1 | 2 | An enzyme that catalyzes the sixth step of glycolysis and thus serves to break down glucose for energy and carbon molecules |
